# New Cardiovascular and Pulmonary Therapeutic Strategies Based on the Angiotensin-Converting Enzyme 2/Angiotensin-(1–7)/Mas Receptor Axis

**DOI:** 10.1155/2012/147825

**Published:** 2012-01-26

**Authors:** Anderson J. Ferreira, Tatiane M. Murça, Rodrigo A. Fraga-Silva, Carlos Henrique Castro, Mohan K. Raizada, Robson A. S. Santos

**Affiliations:** ^1^Department of Morphology, Institute of Biological Sciences, Federal University of Minas Gerais, 31.270-901 Belo Horizonte, MG, Brazil; ^2^Department of Physiology and Biophysics, Institute of Biological Sciences, Federal University of Minas Gerais, 31.270-901 Belo Horizonte, MG, Brazil; ^3^Department of Physiology Sciences, Federal University of Goiás, 74.001-970 Goiânia, GO, Brazil; ^4^Department of Physiology and Functional Genomics, College of Medicine, University of Florida, 32.610 Gainesville, FL, USA

## Abstract

Angiotensin (Ang)-(1–7) is now recognized as a biologically active component of the renin-angiotensin system (RAS). The discovery of the angiotensin-converting enzyme homologue ACE2 revealed important metabolic pathways involved in the Ang-(1–7) synthesis. This enzyme can form Ang-(1–7) from Ang II or less efficiently through hydrolysis of Ang I to Ang-(1–9) with subsequent Ang-(1–7) formation. Additionally, it is well established that the G protein-coupled receptor Mas is a functional ligand site for Ang-(1–7). The axis formed by ACE2/Ang-(1–7)/Mas represents an endogenous counter regulatory pathway within the RAS whose actions are opposite to the vasoconstrictor/proliferative arm of the RAS constituted by ACE/Ang II/AT_1_ receptor. In this review we will discuss recent findings concerning the biological role of the ACE2/Ang-(1–7)/Mas arm in the cardiovascular and pulmonary system. Also, we will highlight the initiatives to develop potential therapeutic strategies based on this axis.

## 1. Introduction

**Figure 1:**
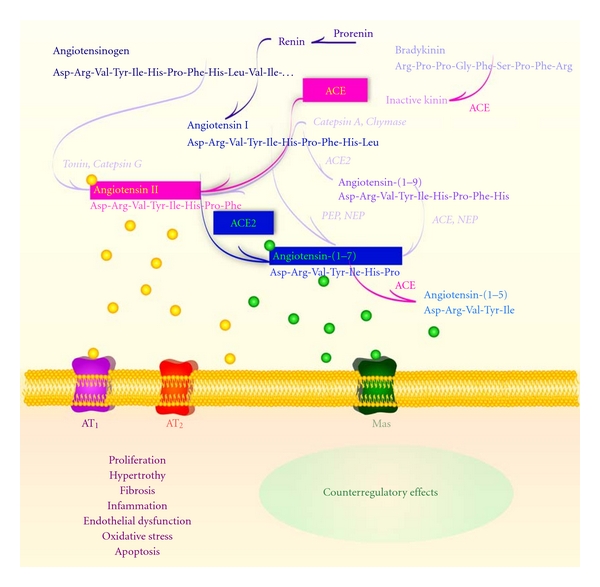
Schematic representation of the renin-angiotensin system (RAS) cascade. The counterregulatory axes of the RAS are composed by ACE/Ang II/AT_1_ and ACE2/Ang-(1–7)/Mas. ACE: angiotensin-converting enzyme; Ang: angiotensin; AT_1_: Ang II type 1 receptor; AT_2_: Ang II type 2 receptor; Mas: Ang-(1–7) receptor; PCP: prolylcarboxypeptidase; PEP: prolyl-endopeptidase; NEP: neutral-endopeptidase 24.11.

The renin-angiotensin system (RAS) plays a key role in several target organs, such as heart, blood vessels, and lungs, exerting a powerful control in the maintenance of the homeostasis [1–4]. This system is activated by the conversion of the angiotensinogen to the inactive peptide angiotensin (Ang) I through the renin action [5]. Subsequently, Ang I is cleaved by the angiotensin-converting enzyme (ACE) generating Ang II [6], the main angiotensin peptide, whose actions are mediated by two G protein-coupled receptors (GPCR), AT_1_ and AT_2 _ [7, 8] (Figure 1). The major physiological functions of Ang II are mediated by AT_1_ receptor [9, 10]. In pathological conditions, activation of this receptor induces deleterious effects, such as vasoconstriction, fibrosis, cellular growth and migration, and fluid retention [11, 12]. On the other hand, Ang II binding to the AT_2_ receptor generally causes opposite effects when compared with those actions mediated by the AT_1_ receptor [13, 14].

Recently, it has been proposed that, in addition to the ACE/Ang II/AT_1_ receptor axis, the RAS possesses a counter regulatory axis composed by ACE2, Ang-(1–7), and Mas receptor (Figure 1). Ang-(1–7) is a biologically active component of the RAS which binds to Mas inducing many beneficial actions, such as vasodilatation, antifibrosis, and antihypertrophic and antiproliferative effects [15–23]. This peptide is produced mainly through the action of ACE2, which has approximately 400-fold less affinity to Ang I than to Ang II [24–26]; thereby, Ang II is the major substrate for Ang-(1–7) synthesis. In fact, the conversion of Ang II to Ang-(1–7) by ACE2 is important to regulate the RAS activity since Ang-(1–7) induces opposite effects to those elicited by Ang II [16–24]. Additionally, ACE2 can form Ang-(1–7) less efficiently through hydrolysis of Ang I to Ang-(1–9) with subsequent Ang-(1–7) formation [24].

The relevance of the RAS is highlighted by the success obtained in therapeutic strategies based on the pharmacological inhibition of this system in cardiovascular and respiratory diseases [27–32]. Blockade of the RAS with ACE inhibitors (ACEi) or AT_1_ receptor antagonists (ARBs) improves the outcomes of patients with hypertension, acute myocardial infarction, and chronic systolic heart failure [33–35]. Furthermore, based on the involvement of the ACE/Ang II/AT_1_ axis in respiratory diseases and the crucial role of the lungs in the RAS metabolism, several studies have reported the contribution of the RAS in lung pathophysiology [28, 30, 31, 36–40]. Importantly, it has been shown that administration of ACEi and ARBs causes substantial increases in plasma Ang-(1–7) levels, leading to the assumption that part of their clinical effects might be mediated by this heptapeptide [41–43]. Indeed, some effects of ACEi and ARBs can be blocked or attenuated by A-779, a Mas antagonist, confirming the role of Ang-(1–7) in the actions of these compounds [44]. The beneficial effects of Ang-(1–7), as well as its likely participation in the effects of the ACEi and ARBs, represent evidences for the potential of the ACE2/Ang-(1-7)/Mas axis as a therapeutic target.

In this review, we will focus on the recent findings related to the pathophysiology actions of the ACE2/Ang-(1–7)/Mas axis in the cardiovascular and respiratory system. Also, we will discuss the promising initiatives to develop new therapeutic strategies based on this axis to treat pathological conditions.

## 2. Cardiac ACE2/Ang-(1–7)/Mas Axis

The heart is one of the most important targets for the actions of the ACE2/Ang-(1–7)/Mas axis. In the heart, ACE2 is expressed in the endothelium [45], myofibroblasts [46], cardiomyocytes, and fibroblasts [47, 48]. Classical pharmacotherapeutic agents used to treat heart failure, including ACEi, ARBs, and aldosterone receptor blockers, increase ACE2 activity and/or expression, indicating its importance in the cardiac diseases establishment and progression [49–51].

Additionally, pharmacological and genetic (transgenic animals and gene transfer) approaches have evidenced the significance of ACE2 in cardiac pathologies. Despite some controversies concerning the consequences of the ACE2 deficiency, in general, evidences indicate a protective role of ACE2 in the heart [48, 52–57]. Crackower and colleagues [52] were the first to demonstrate that genetic ablation of ACE2 results in severe blood-pressure-independent systolic impairment. Also, disruption of ACE2 was able to accelerate cardiac hypertrophy and shortened the transition period to heart failure in response to pressure overload by increasing local Ang II [54]. Recently, it has been demonstrated that loss of ACE2 enhances the susceptibility to myocardial infarction, with increased mortality, infarct expansion and adverse ventricular remodeling [56]. In keeping with these genetic findings, pharmacological inhibition of ACE2 exacerbated cardiac hypertrophy and fibrosis in Ren-2 hypertensive rats [58]. On the other hand, cardiac overexpression of ACE2 prevented hypertension-induced cardiac hypertrophy and fibrosis in spontaneously hypertensive rats (SHR) and in Ang-II-infused rats [59, 60]. Indeed, transfection of Lenti-ACE2 (lentivirus containing ACE2 cDNA) or Ad-ACE2 (recombinant adenovirus carrying the murine ACE2) into the surrounding area of the infarcted myocardium was protective against pathological remodeling and cardiac systolic dysfunction in a rat model of myocardial infarction [61, 62]. This effect was associated with decreased expression of ACE and Ang II and increased expression of Ang-(1–7) [62]. Collectively, these observations reveal that ACE2 effectively plays a protective role in the cardiac structure and function.

Since the discovery of Ang-(1–7) in the late 1980s [63, 64], several studies have demonstrated important effects of this peptide in hearts. The presence of Ang-(1–7) and its receptor Mas in the heart [65, 66] and the ability of this organ to produce Ang-(1–7) [55, 67] are evidences of the role of this peptide in cardiac tissues. Functionally, Ang-(1–7) induces an antiarrhythmogenic effect against ischemia/reperfusion injuries in rats [17, 68] as well as prevents atrial tachycardia and fibrillation in rats and dogs [69, 70]. Treatment with Ang-(1–7) improved the coronary perfusion and cardiac function in rats after myocardial infarction [71] and after ischemia/reperfusion injury [72]. Increases in circulating Ang-(1–7) levels in transgenic rats reduced the cardiac hypertrophy [17] and fibrosis [20, 22] induced by isoproterenol administration. These effects are apparently independent of changes in blood pressure since Grobe and colleagues [18] have demonstrated that the antifibrotic and antihypertrophic actions of Ang-(1–7) are still observed in Ang-II-infused hypertensive rats. Local overexpression of Ang-(1–7) in hearts of mice and rats improved the myocardial contractility and prevented the isoproterenol- and hypertension-induced cardiac remodeling [19, 21]. Altogether, these findings support a direct effect of Ang-(1-7) in the heart.

Further evidence for the role of Ang-(1–7)/Mas in the pathophysiology of the heart came from experimental protocols utilizing mice with genetic deficiency of Mas. They revealed that the cardiac function is impaired in Mas knockout mice likely due to the increased extracellular matrix proteins deposition in the heart [66, 73]. This profibrotic phenotype may be related to changes in matrix metalloproteinases (MMPs) and tissue inhibitors of metalloproteinases (TIMPs) levels and/or activities [74, 75].

Although further elucidations regarding the signaling pathways involved in Mas activation are necessary, some mechanisms have been proposed. Overexpression of Ang-(1–7) in hearts of rats causes an improvement in the [Ca^2+^] handling in cardiomyocytes and increases the expression of SERCA2a [21]. In keeping with these results, cardiomyocytes from Mas-deficient mice present slower [Ca^2+^]_i_ transients accompanied by a lower Ca^2+^ ATPase expression in the sarcoplasmic reticulum [66, 76]. Although acute Ang-(1–7) treatment failed to alter Ca^2+^ handling in ventricular myocytes of rats [76], these findings suggest an important role of the Ang-(1–7)/Mas in the long-term maintenance of the Ca^2+^ homeostasis in the heart.

One of the mechanisms by which Ang-(1–7) plays its effects in the heart is stimulating the nitric oxide (NO) production. Indeed, it has been demonstrated that Ang-(1–7) via Mas increases the synthesis of NO through a mechanism involving the activation of the endothelial NO synthase (eNOS). These effects were abolished by A-779 and are absent in cardiomyocytes from Mas-deficient mice [76]. Recently, Gomes et al. [77] found that the treatment of isolated cardiomyocytes of rats with Ang-(1–7) efficiently prevents the Ang-II-induced hypertrophy by modulating the calcineurin/NFAT signaling cascade. These effects were blocked by NO synthase inhibition and by guanylyl cyclase inhibitors, indicating that these effects are mediated by the NO/cGMP pathway.

Also, Ang-(1–7) inhibits serum-stimulated mitogen-activated protein kinase (MAPK) activation in cardiac myocytes [78] and prevents the Ang-II-mediated phosphorylation of ERK1/2 and Rho kinase in hearts in a dose-dependent manner [79]. In line with these data, activation of endogenous ACE2 significantly reduced the phosphorylation of ERK1/2 in hearts of hypertensive rats (SHRs) [48]. However, Mercure et al. [19] reported that overexpression of Ang-(1–7) in hearts of rats decreases the Ang-II-induced phosphorylation of c-Src and p38 kinase, whereas the increase in ERK1/2 phosphorylation was unaffected by the expression of the transgene, thereby suggesting a selective effect of Ang-(1–7) on intracellular signaling pathways related to cardiac remodeling.

Overall, these data reveal a key role of the ACE2/Ang-(1–7)/Mas axis in the pathophysiology of the cardiac structure and function. Activation of this axis might be an important strategy to develop a new generation of cardiovascular therapeutic agents against cardiac dysfunction and pathological remodeling of the heart.

## 3. Vascular ACE2/Ang-(1–7)/Mas Axis

Early studies have reported the endothelium as the major site for generation [67] and metabolism [41] of Ang-(1–7). In addition to Ang-(1–7), endothelial cells also express ACE2 and Mas [80, 81]. Thus, now it is recognized that the ACE2/Ang-(1–7)/Mas axis is present in vascular endothelial cells and modulates its function promoting vasorelaxation [82], reduction of the oxidative stress [83, 84], and antiproliferative effects [85, 86].

The vasodilatory actions of Ang-(1–7) have been reported in many studies in several vascular beds and preparations, including mouse [16, 23] and rat [15] aortic rings, canine [87] and porcine [88] coronary arteries, canine middle cerebral artery [89], porcine piglet pial arterioles [90], feline mesenteric vascular bed [91], rabbit renal afferent arterioles [92], and mesenteric microvessels of normotensive [93] and hypertensive [94] rats. Vascular Ang-(1–7) actions are still controversial in human. For example, it has been shown that Ang-(1–7) causes vasodilation in forearm circulation of normotensive subjects and patients with essential hypertension [95] while other studies were unable to report any significant effect of Ang-(1–7) in the same vascular territory in ACEi-treated patients [43].

The Mas receptor is critically involved in the vascular effects of Ang-(1–7). In fact, many of these actions are completely abolished by A-779 or partially blocked by this antagonist [3, 86, 96]. Importantly, the endothelium-dependent relaxation induced by Ang-(1–7) in mouse aortic rings is absent in vessels derived from Mas-knockout mice [16]. However, other studies have shown that Ang-(1–7) also interacts with ACE, AT_1_, and AT_2_-like receptors, suggesting the existence of additional sites of interaction for Ang-(1–7) [3, 97, 98]. Indeed, Silva et al. [99] reported evidence for the presence of a distinct subtype of Ang-(1–7) receptor sensible to D-pro^7^-Ang-(1–7), a second Mas antagonist, but not to A-779 in aortas of Sprague-Dawley rats.

The vascular effects of Ang-(1–7) are endothelium dependent and involve the production of vasodilator products, such as prostanoids, NO, and endothelium-derived hyperpolarizing factor (EDHF) [16, 81, 100]. Pinheiro and coworkers [101] found that Ang-(1–7) promotes an increase in NO release in Mas-transfected chinese hamster ovary (CHO) cells [101]. Furthermore, short-term infusion of Ang-(1–7) improved the endothelial function by a mechanism involving NO release in rats [102]. Mas deletion resulted in endothelial dysfunction associated with an unbalance between NO and oxidative stress [83]. Also, Mas activation by Ang-(1–7) in human endothelial cells stimulated eNOS phosphorylation/activation via the Akt-dependent pathway [81]. Other mechanisms appear to be involved in the Ang-(1–7) vascular actions. Roks et al. [103] have shown that Ang-(1–7) inhibits the vasoconstriction induced by Ang II in human internal mammary arteries, thereby suggesting that Ang-(1–7) can regulate the Ang II effects [103]. In fact, Ang-(1–7) negatively modulates the Ang II type 1 receptor-mediated activation of c-Src, and its downstream targets ERK1/2 and NAD(P)H oxidase [104]. The counterregulatory action of Ang-(1–7) on Ang II signaling has been also observed in cardiomyocytes [77], vascular smooth muscle cells [105], and fibroblasts [106]. Additionally, an interaction between Mas and bradykinin (Bk) type 2 (B_2_) receptors may modulate some of the Ang-(1–7) effects in blood vessels [107]. Indeed, it has been demonstrated that Ang-(1–7) potentiates the vasodilator and hypotensive effects of Bk in several vascular beds [93, 108–110].

**Figure 2:**
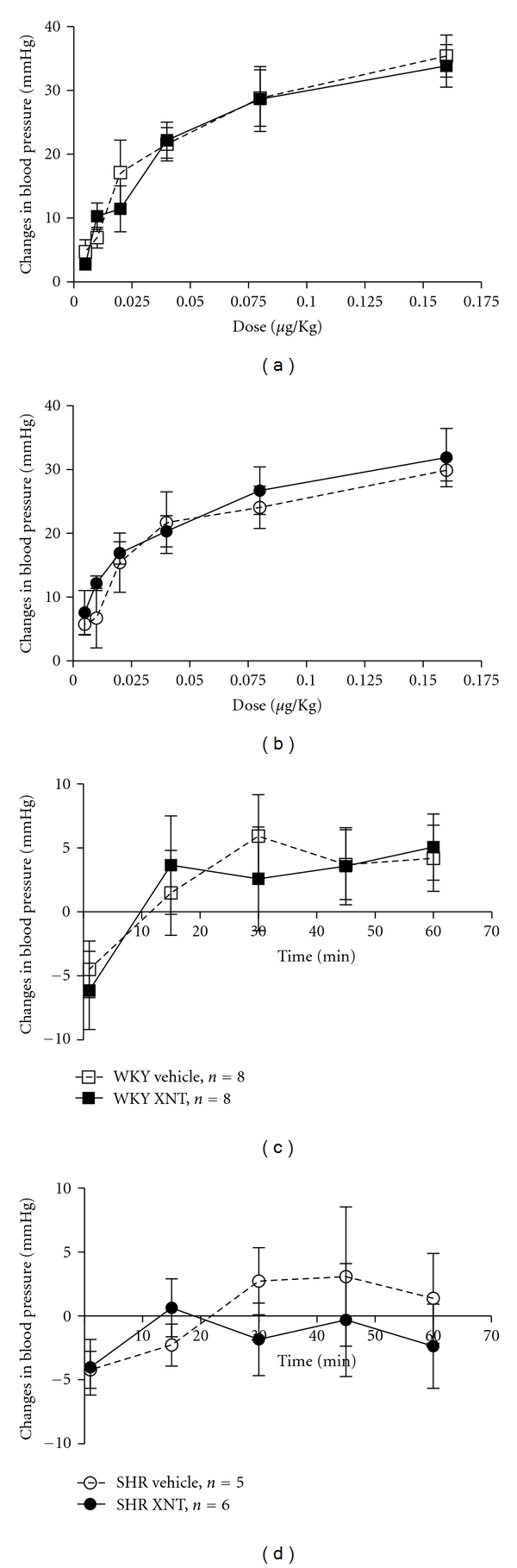
Effects of Ang II and Losartan on arterial blood pressure of rats chronically treated with XNT. The responses to increasing doses of Ang II were similar in vehicle- and XNT-treated (a) normotensive (Wistar-Kyoto rats—WKY) and (b) hypertensive (spontaneously hypertensive rats—SHR) rats. Likewise, the response to Losartan (0.25 mg/kg) was similar in vehicle- and XNT-treated (c) normotensive (WKY) and (d) hypertensive (SHRs) rats. The blood pressure was measured through a catheter inserted into the carotid artery and Ang II and Losartan were administrated *in bolus* using the jugular vein.

As the major enzyme involved in Ang-(1–7) formation, ACE2 has also a crucial role in vessels. Lovren et al. [111] have demonstrated that ACE2 ameliorates the endothelial homeostasis via a mechanism involving reduction of the reactive oxygen species production [111]. Of note, this effect was attenuated by A-779 [111]. Moreover, overexpression of ACE2 in vessels of hypertensive rats resulted in reduction in the arterial blood pressure and improvement of the endothelial function associated with increased circulating Ang-(1–7) levels [112]. Overall, these data indicate that the beneficial effects of ACE2 are, at least in part, mediated by Ang-(1–7). Recently, we have demonstrated that activation of endogenous ACE2 causes a dose-dependent hypotensive effect in normotensive and hypertensive rats [113]. Also, the response to Bk administration was augmented in rats chronically treated with XNT, an ACE2 activator [113]. However, we were unable to demonstrate any significant effect of XNT on blood pressure in response to the administration of Ang II or Losartan in normotensive and hypertensive rats (Figure 2).

## 4. Pulmonary ACE2/Ang-(1–7)/Mas Axis

In the past few years, the participation of the ACE2/Ang-(1–7)/Mas axis in the establishment and progression of pulmonary diseases has become evident. Indeed, the important role of the RAS in the lung pathophysiology and the side effects and pulmonary toxicity induced by the ACEi raised the interest to evaluate the activation of the ACE2/Ang-(1–7)/Mas axis as an alternative target to treat pulmonary pathologies. Thus, it has been reported beneficial outcomes induced by the activation of this axis in animal models of acute respiratory distress syndrome (ARDS), pulmonary hypertension (PH), fibrosis, and lung cancer [31, 37, 114–117]. These studies pointed out that the imbalance between the ACE/Ang II/AT_1_ and the ACE2/Ang-(1–7)/Mas axes of the RAS might be relevant in lung diseases. Taking into account that systemic hypotension is an important limitation to the use of ACEi and ARBs in pulmonary patients, therapies based on the ACE2/Ang-(1–7)/Mas axis emerge as a safe and efficient approach since studies using the ACE2 activator XNT or ACE2 gene transfer have shown that these strategies induce beneficial pulmonary outcome without changes in systemic blood pressure in rats and mice [39, 117, 118].

Imai and colleagues [37] demonstrated the role of ACE2 in ARDS pathogenesis. They found that a more severe ARDS was reached in ACE2 knockout mice, and this phenotype was reversed by double genetic deletion of the ACE2 and ACE genes or by the treatment with recombinant human ACE2 (rhACE2). Furthermore, Ang II levels were related to the severity of the lung injury. Of note, ACE2 is widely expressed in the pulmonary endothelium, vasculature, and pneumocytes [119, 120]. Also, rhACE2 inhibited the increase of Ang II and TNF-*α* levels, attenuated the arterial hypoxemia and PH, and ameliorated the distribution of the pulmonary blood flow in lipopolysaccharide-induced lung injury in piglets [121]. Therefore, these studies suggest that ACE2 is a suitable target to arrest the development of ARDS in patients at risk.

The stimulation of the ACE2/Ang-(1–7)/Mas axis has been successful used to prevent and reverse PH and fibrosis in animals. ACE2 activation using the compound XNT or induction of ACE2 overexpression by gene transfer efficiently prevented and, more importantly, reversed the increase of the right systolic ventricular pressure (RSVP), pulmonary fibrosis, imbalance of the RAS, and inflammation in animals (rats and mice) with PH induced by monocrotaline (MCT) or in rats with pulmonary fibrosis caused by bleomycin treatment [39, 117, 118]. In keeping with these findings, Ang-(1–7) gene transfer into the lungs triggered similar protective actions in MCT-treated rats [39]. In addition, Ang-(1–7) via Mas prevented the apoptosis of alveolar epithelial cells and the Jun N-terminal kinase (JNK) activation induced by bleomycin [122]. The involvement of the Ang-(1–7)/Mas in PH was further evidenced by the observation that the XNT effects are blocked by A-779 [117]. Furthermore, in both lung specimens from patients with idiopathic pulmonary fibrosis and from animals with bleomycin-induced pulmonary fibrosis were reported a reduction in mRNA, protein, and activity of ACE2 with a reciprocal increase in Ang II level [116].

A growing body of studies has focused on the relevance of the ACE2/Ang-(1–7)/Mas axis in the pulmonary cancer pathophysiology. The protein expression of ACE2 is reduced in non-small-cell lung carcinoma (NSCLC) along with an increase in Ang II levels. Moreover, overexpression of ACE2 in cultured A549 lung cancer cells and in human lung cancer xenografs inhibited the cell growth and the vascular endothelial growth factor-a (VEGFa) expression induced by Ang II [123, 124]. Gallagher and Tallant [125] evaluated the effects of several angiotensin peptides [Ang I, Ang II, Ang-(2–8), Ang-(3–8), and Ang-(3–7)] in SK-LU-1 cancer cells growth, and only Ang-(1–7) showed significant attenuation of the DNA synthesis and proliferation. The antiproliferative effect of Ang-(1–7) was mediated by its receptor Mas and inhibition of the ERK1/2 pathway. Neither the blockage of AT_1_ nor AT_2_ succeeded in inhibiting the action of Ang-(1–7). In keeping with these data, the antiproliferative effect of Ang-(1–7) was observed in human A549 lung tumor xenograft growth along with a marked decrease in the vessel density in mice through a mechanism involving cyclooxygenase-2 (COX-2) [126, 127]. Of note, in a nonrandomized phase I clinical trial conducted by Petty and colleagues [38], subcutaneous injections of Ang-(1–7) were administered in 18 patients with advanced solid tumors refractory to standard therapy. Despite the mild adverse effects observed with the Ang-(1–7) treatment, generally it was well tolerated. There were no treatment-related deaths. Clinical benefits were observed in 27% of the patients. Altogether, these studies provide insights into the involvement of the ACE2/Ang-(1–7)/Mas axis in lung cancer.

## 5. Pharmacological Therapeutic Strategies Based on the ACE2/Ang-(1–7)/Mas Axis

Many advances have been achieved regarding the therapeutic regulation of the RAS. Current therapies based on the modulation of the RAS include the ACEi, ARBs, and renin inhibitors. In general, these drugs prevent or reverse endothelial dysfunction and atherosclerosis, reduce cardiovascular mortality and morbidity of patients with coronary artery disease, and hold antihypertensive effects [128].

**Figure 3:**
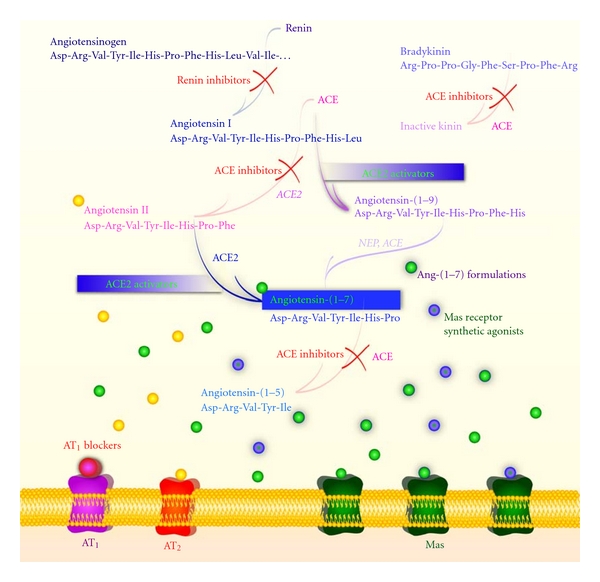
Schematic diagram showing the therapeutic strategies to modulate the activity of the renin-angiotensin system (RAS). In addition to the classical RAS blockers, that is, ACE inhibitors and AT_1_ receptor blockers, the figure highlights the renin inhibitors, the Ang-(1–7) formulations [HP*β*CD/Ang-(1-7) and cyclic Ang-(1-7)], the synthetic Mas receptor agonists (AVE 0991 and CGEN-856S), and the ACE2 activator (XNT). ACE: angiotensin-converting enzyme; AT_1_: Ang II type 1 receptor; AT_2_: Ang II type 2 receptor; Mas: Ang-(1–7) receptor; NEP: neutral-endopeptidase 24.11.

Classically, the mechanisms of action of the ACEi and ARBs involve the blockade of the synthesis and actions of Ang II, respectively. However, the RAS is a complex hormonal system and, consequently, other mechanisms are likely implicated in the actions of these drugs [42, 86, 129]. They cause substantial increase in plasma levels of Ang-(1–7), leading to the assumption that their clinical effects might be partly mediated by this heptapeptide [42, 130]. Indeed, a variety of effects of the ACEi and ARBs can be abolished or attenuated by Mas antagonism, confirming the role of Ang-(1–7) in the actions of these compounds [129, 131]. The beneficial effects of Ang-(1–7) as well as its likely involvement in the effects of the ACEi and ARBs represent a strong evidence for the therapeutic potential of the activation of the ACE2/Ang-(1–7)/Mas axis (Figure 3).

### 5.1. Ang-(1–7) Formulations

The beneficial effects of Ang-(1–7) are well known; however, the therapeutic utilization of this peptide is limited due to its unfavorable pharmacokinetic properties. Ang-(1–7) has a short half-life (approximately 10 seconds) since it is rapidly cleaved by peptidases [132]. Furthermore, Ang-(1–7) is degraded during its passage through the gastrointestinal tract when orally administrated. Thus, new strategies are crucial to make feasible the clinical application of Ang-(1–7).

Recently, a formulation based on the Ang-(1–7) included into hydroxypropyl *β*-cyclodextrin [HP*β*CD/Ang-(1–7)] was developed by Lula and colleagues [133]. Cyclodextrins are pharmaceutical tools used for design and evaluation of drug formulations, and they enhance the drug stability and absorption across biological barriers and offer gastric protection [134]. The amphiphilic character of cyclodextrins allows the possibility of formation of supramolecular inclusion complexes stabilized by noncovalent interactions with a variety of guest molecules [133, 134]. In this regard, the formulation HP*β*CD/Ang-(1–7) allowed the oral administration of Ang-(1–7). Pharmacokinetic and functional studies showed that oral HP*β*CD/Ang-(1–7) administration significantly increases plasma Ang-(1–7) levels and promotes an antithrombotic effect that was blunted in Mas deficient mice [135]. Marques and colleagues [136] have found that chronic oral administration of HP*β*CD/Ang-(1–7) significantly attenuates the heart function impairment and cardiac remodeling induced by isoproterenol treatment and myocardial infarction in rats [136].

In addition, liposomal delivery systems represent an alternative method to administer Ang-(1–7) [137]. Administration of liposomes containing Ang-(1–7) in rats led to prolonged hypotensive effect for several days in contrast to the response observed when the free peptide was used [137, 138].

A strategy used to protect the Ang-(1–7) against proteolytic degradation was proposed by Kluskens and coworkers [139]. Using the ability of prokaryotes to cyclize peptides, they synthesized a cyclic Ang-(1–7) derivative [thioether-bridged Ang-(1–7)] which presented an increased stability in homogenates of different organs and plasma and enhanced the Ang-(1–7) bioavailability in rats [139]. Furthermore, cyclized Ang-(1–7) induced a relaxation in precontracted aorta rings of rats which was blocked by the Ang-(1–7) receptor antagonist D-Pro^7^-Ang-(1–7), providing evidence that cyclized Ang-(1–7) also interacts with Mas [139].

### 5.2. Synthetic Mas Receptor Agonists

AVE 0991 was the first nonpeptide synthetic compound developed with the intention of stimulating the Mas receptor. This compound mimics the Ang-(1–7) effects in several organs such as vessels [140, 141], kidney [101], and heart [142, 143]. Similar to Ang-(1–7), AVE 0991 induced a vasodilation effect which was absent in aortic rings of Mas-deficient mice [140]. Moreover, its effects in aortic rings were blocked by the two Ang-(1–7) receptor antagonists, A-779 and D-Pro^7^-Ang-(1–7) [140]. AVE 0991 potentiated the acetylcholine-induced vasodilation in conscious normotensive rats, and this effect was abolished by A-779 and L-NAME [102]. Similarly, it was able to increase the hypotensive effect of Bk in normotensive rats, and A-779 also blocked this effect [107]. Ferreira et al. [142, 143] reported that AVE 0991 protects the heart against cardiac dysfunction and remodeling caused by isoproterenol treatment or by myocardial infarction in rats [142, 143]. In *Mas*-transfected cells, AVE 0991 induced NO release which was blunted by A-779 and not by AT_2_ or AT_1_ antagonists [101]. All these data support the concept that AVE 0991 is an Ang-(1–7) mimetic and that its actions are mediated by the interaction with Mas.

Using a computational discovery platform for predicting novel naturally occurring peptides that may activate GPCR, two novel peptides, designated as CGEN-856 and CGEN-857, with amino acid sequence unrelated to angiotensin peptides, were found to display high specificity for Mas [23]. These peptides elicited Ca^+2^ influx in CHO cells overexpressing Mas without any activity in AT_1_ or AT_2_ receptors [144]. CGEN-856S, a derivative of the CGEN-856 peptide, induced beneficial cardiovascular effects similar to those caused by Ang-(1–7) [23]. This compound competes with Ang-(1–7) for the same bind site in *Mas*-transfected cells. Furthermore, similar to Ang-(1–7), CGEN-856S produced a vasodilation effect which was absence in Mas-deficient mice, indicating that this compound also acts via Mas [23]. This was confirmed by the inhibition of the CGEN-856S effects by the Mas antagonist A-779. Importantly, Savergnini et al. [23] showed that CGEN-856S promotes antiarrhythmogenic effects and produces a small dose-dependent decrease in arterial pressure of conscious SHR [23].

### 5.3. ACE2 Activators

A new approach addressing the therapeutic potential of the activation of the ACE2/Ang-(1–7)/Mas axis was proposed by Hernández Prada et al. [113]. Based on the crystal structure of ACE2 and using a virtual screening strategy, it was identified small molecules that may interact with this enzyme leading to changes in its conformation and, consequently, enhancing its activity [113]. Thus, the ACE2 activator, namely XNT, was identified and its administration in SHR decreased blood pressure, induced an improvement in cardiac function, and reversed the myocardial and perivascular fibrosis observed in these animals [48, 113]. The beneficial effects of XNT were also observed in rats with PH induced by MCT [117]. Furthermore, this compound attenuated the thrombus formation and reduced the platelet attachment to vessels in hypertensive rats [145].

It appears that the pharmacological activation of ACE2 promotes its beneficial effects due to an increased Ang-(1–7) production with concomitant degradation of Ang II. In fact, coadministration of A-779 abolished the protective effects of XNT on PH [117]. In addition, the antifibrotic effect of XNT observed in hearts of SHR was associated with increases in cardiac Ang-(1–7) expression [48]. However, it is also pertinent to point out that off-target effects of XNT on these beneficial outcomes cannot be ruled out at the present time.

## 6. Conclusions

The complexity of the RAS is far beyond we could suspect few years ago. There is growing evidence that changes in the novel components of the RAS [Ang-(1–7), ACE2, and Mas] may take part of the establishment and progression of cardiovascular and respiratory diseases. Importantly, these new components of the RAS, due to their counter regulatory actions, are candidates to serve as a concept to develop new cardiovascular and respiratory drugs.
